# Supplementary material for: Monoclonal antibodies targeting sites in respiratory syncytial virus attachment G protein provide protection against RSV-A and RSV-B in mice
Source: Nat Commun. 2024 Apr 4;15:2900. doi: 10.1038/s41467-024-47146-2 (PMC10994933; doi:10.1038/s41467-024-47146-2)
Supplement: Supplementary file 1 — Supplementary Information [file 41467_2024_47146_MOESM1_ESM.pdf]

Figure S1

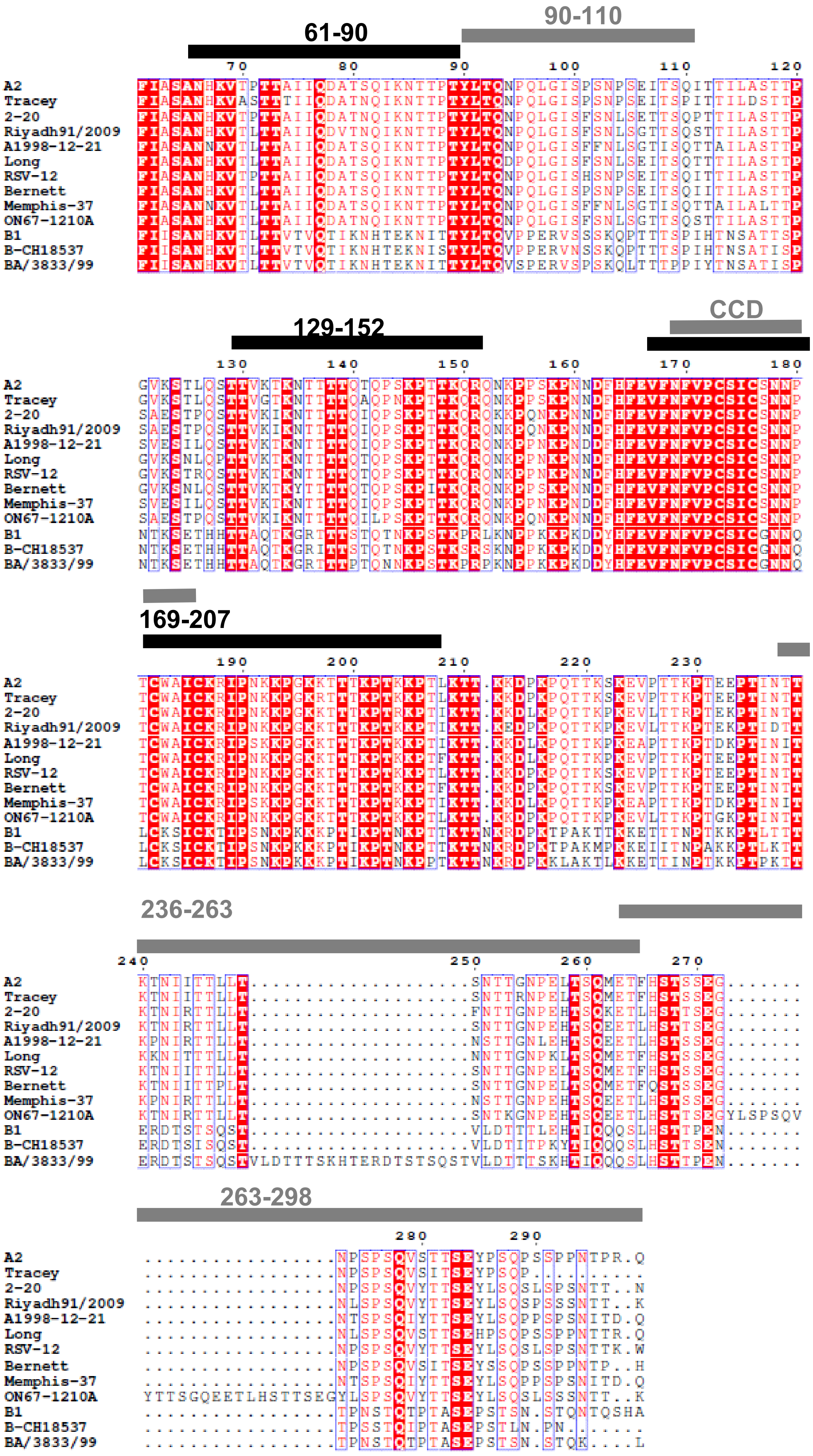

Figure S1. RSV G amino acid sequence alignment of the phylogeny, subtype sequence alignment. Sequence alignment of RSV G ectodomain from diverse RSV strains. Strains include A2, A-TX-Tracey, A2001-2-20, A/Riyadh/2009, A1998-12-21, Long, RSV-12, A-Bernett-61, Memphis-37, ON67-1210A, B1, B-CH18537, and BA/3833/99. Peptides in RSV-G that were used for antibody binding analysis are displayed in black and gray lines. Plotting identities and similarities in alignment sequence with red color-boxes.

Figure S2

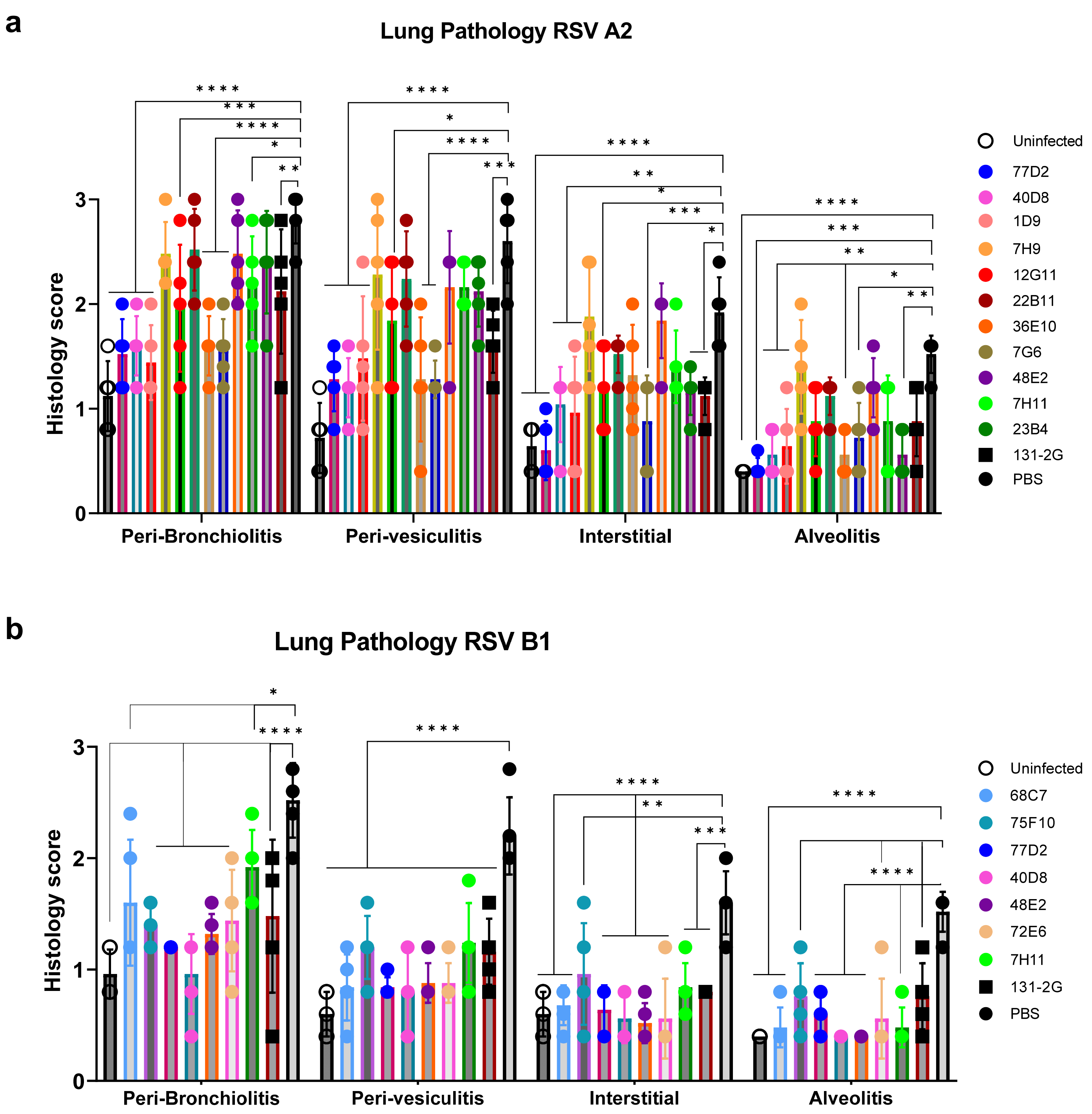

Figure S2: Histopathology analyses in lungs from MAb treated mice following RSV challenge. Lung tissues were collected at 5 days post-challenge with RSV-A2 (A) or RSV-B1 (B) and stained with hematoxylin and eosin. Individual lungs were scored for pulmonary inflammation: peri-bronchiolitis (inflammatory cell infiltration around the bronchioles), peri-vasculitis (inflammatory cell infiltration around the small blood vessels), interstitial pneumonia (inflammatory cell infiltration and thickening of alveolar walls), and alveolitis (cells within the alveolar spaces). Slides were scored blindly using a 0-3 severity scale. Results are presented as mean ± SEM. One-way ANOVA multiple comparisons test was performed in GraphPad Prism; *** p<0.0001, ***p<0.001, **p<0.01, *p<0.05.

Figure S3

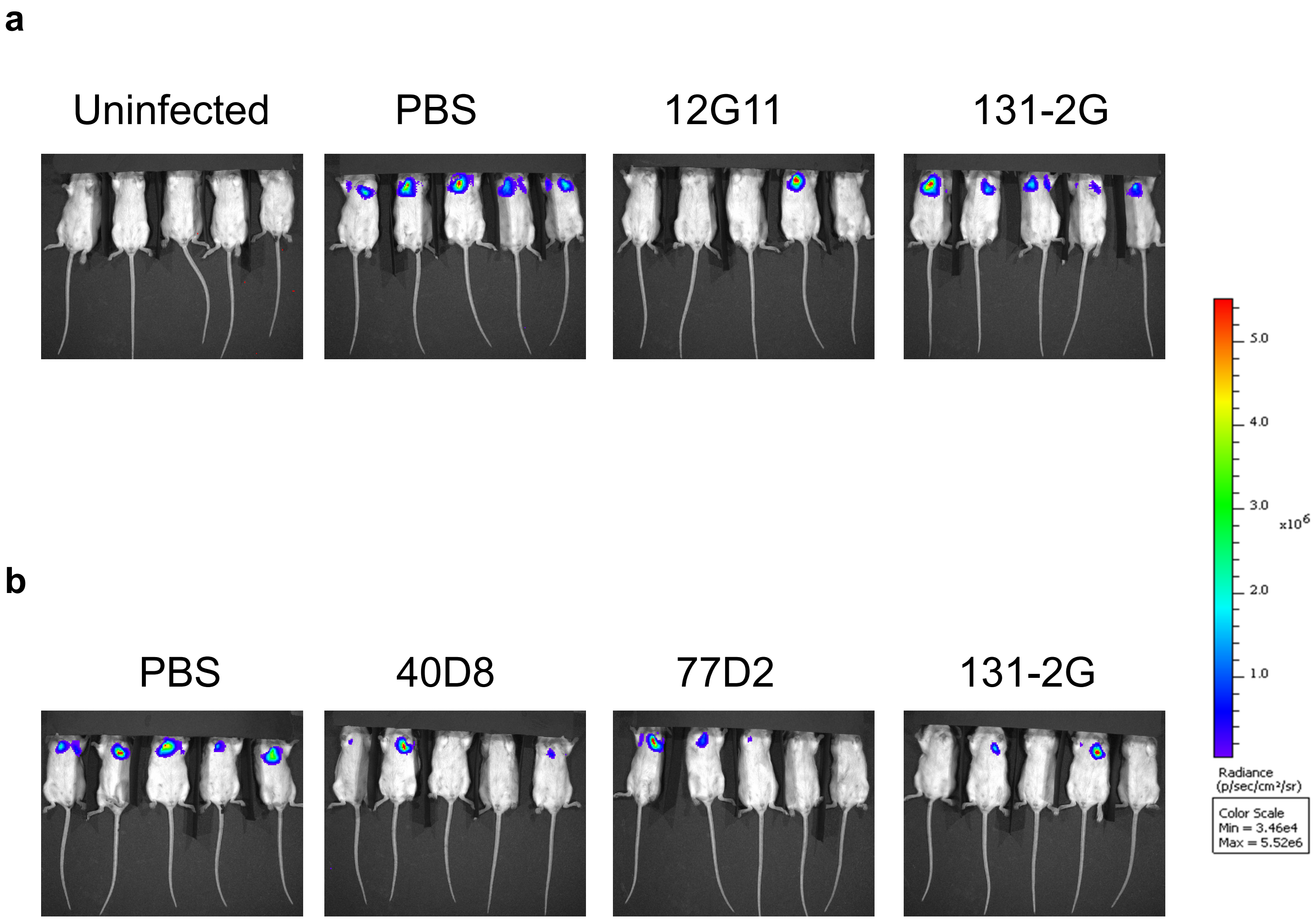

Figure S3: Representative *in vivo* imaging of RSV infection in mice. BALB/c mice were infected intranasally with 10e6 pfu of RSV rA2-line19F-FFL (a) or RSV-B1-FFL (b) virus strain. A group of five mice were kept uninfected and serve as the background control. Representative images of *in vivo* imaging of lungs on day 5 post-challenge from selected group of RSV infected mice with either RSV-A2-FFL (a) or RSV-B1-FFL (b).

Figure S4

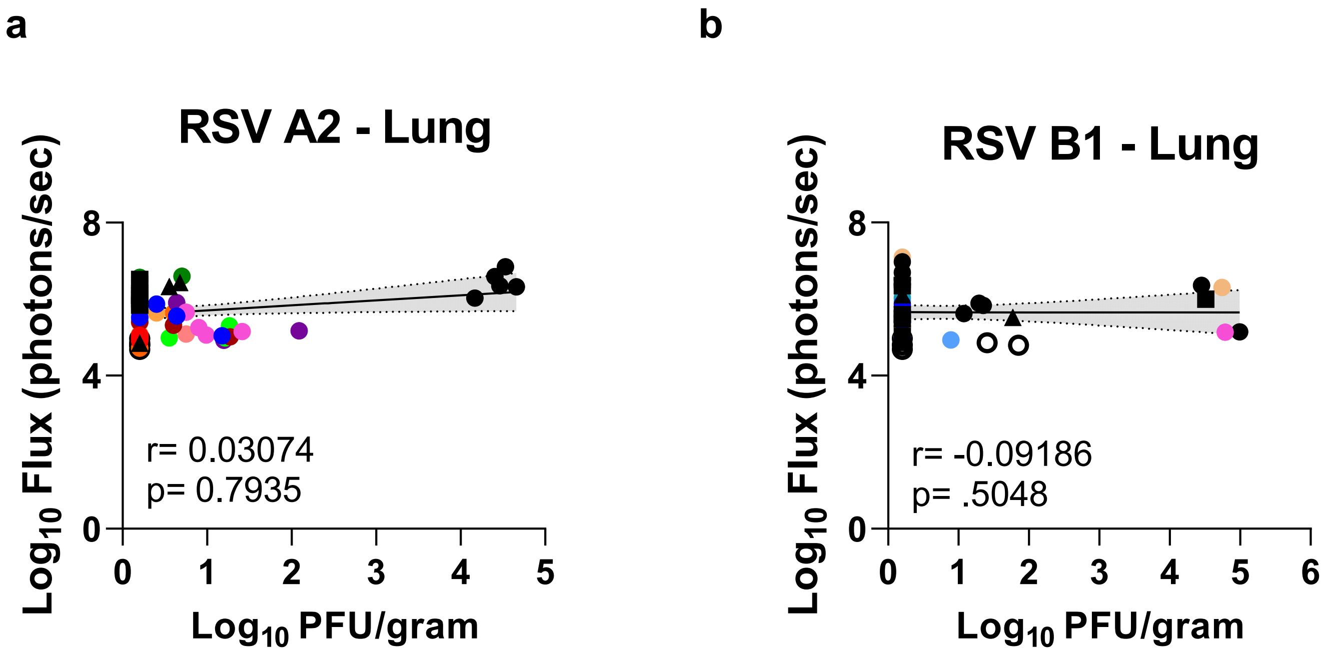

Figure S4: Relationship of lung pathology and lung fluxes or viral load titer on day 5 post-challenge with either RSV-A2 or RSV-B1. Correlation of lung infectious viral load measured by plaque assay versus bioluminescence flux signal in the infected lungs on day 5 post-challenge with either RSV-A2-FFL (a) or RSV-B1-FFL (b) for all MAb treated BALB/c mice. Correlations show Spearman correlation coefficient (r) and two-tailed p values for all samples. The black line in the scatter plots depict the linear fit with shaded area showing 95% confidence interval.

Table S1: RSV Immunogen used to generate MAbs and RSV neutralization titers measured by PRNT assay (in the presence of GPC)

MAb ID	RSV Immunogen	Endpoint Titer	
		RSV-A2	RSV-B1
68C7	REG-B1	0.00	0.00
12F12	REG-A2	0.00	0.00
69C1	REG-B1	0.00	0.00
75F10	REG-B1	0.00	0.00
77D2	REG-B1	0.00	0.00
40D8	REG-A2	0.00	0.00
1D9	REG-A2	0.00	0.00
7H9	REG-A2	0.00	0.00
12G11	REG-A2	0.00	0.00
22B11	REG-A2	0.00	0.00
36E10	REG-A2	0.00	0.00
7C6	REG-A2	0.00	0.00
7G6	REG-A2	0.00	0.00
48E2	REG-A2	0.00	0.00
72E6	REG-B1	0.00	0.00
7H11	REG-A2	0.00	0.00
23B4	REG-A2	0.00	0.00
131-2G	-	0.00	0.00
Negative Control (Anti-HIV MAb 2D7)	-	0.00	0.00
Rabbit anti-REG-A2	REG-A2	49145.46	906.37
