# Supplementary material for: Monoclonal antibodies targeting sites in respiratory syncytial virus attachment G protein provide protection against RSV-A and RSV-B in mice
Source: Nat Commun. 2024 Apr 4;15:2900. doi: 10.1038/s41467-024-47146-2 (PMC10994933; doi:10.1038/s41467-024-47146-2)
Supplement: Supplementary file 3 — Reporting Summary [file 41467_2024_47146_MOESM3_ESM.pdf]

nature portfolio

Corresponding author(s): Surender Khurana

Last updated by author(s): Feb 27, 2024

Reporting Summary

Nature Portfolio wishes to improve the reproducibility of the work that we publish. This form provides structure for consistency and transparency in reporting. For further information on Nature Portfolio policies, see our Editorial Policies and the Editorial Policy Checklist.

Statistics

For all statistical analyses, confirm that the following items are present in the figure legend, table legend, main text, or Methods section.

n/a	Confirmed	
☐	☒	The exact sample size (*n*) for each experimental group/condition, given as a discrete number and unit of measurement
☐	☒	A statement on whether measurements were taken from distinct samples or whether the same sample was measured repeatedly
☐	☒	The statistical test(s) used AND whether they are one- or two-sided<br>*Only common tests should be described solely by name; describe more complex techniques in the Methods section.*
☐	☒	A description of all covariates tested
☐	☒	A description of any assumptions or corrections, such as tests of normality and adjustment for multiple comparisons
☐	☒	A full description of the statistical parameters including central tendency (e.g. means) or other basic estimates (e.g. regression coefficient) AND variation (e.g. standard deviation) or associated estimates of uncertainty (e.g. confidence intervals)
☐	☒	For null hypothesis testing, the test statistic (e.g. *F*, *t*, *r*) with confidence intervals, effect sizes, degrees of freedom and *P* value noted<br>*Give P values as exact values whenever suitable.*
☒	☐	For Bayesian analysis, information on the choice of priors and Markov chain Monte Carlo settings
☒	☐	For hierarchical and complex designs, identification of the appropriate level for tests and full reporting of outcomes
☐	☒	Estimates of effect sizes (e.g. Cohen's *d*, Pearson's *r*), indicating how they were calculated

Our web collection on statistics for biologists contains articles on many of the points above.

Software and code

Policy information about availability of computer code

Data collection	All experimental data was collected in MS Excel version 16.57.
Data analysis	All experimental data were plotted and statistical analysis were performed using Prism 9.3.1 (GraphPad Software).

For manuscripts utilizing custom algorithms or software that are central to the research but not yet described in published literature, software must be made available to editors and reviewers. We strongly encourage code deposition in a community repository (e.g. GitHub). See the Nature Portfolio guidelines for submitting code & software for further information.

Data

Policy information about availability of data

All manuscripts must include a data availability statement. This statement should provide the following information, where applicable:

- Accession codes, unique identifiers, or web links for publicly available datasets
- A description of any restrictions on data availability
- For clinical datasets or third party data, please ensure that the statement adheres to our policy

All data are shown in the manuscript figures and supplementary information. The complete dataset for this study are provided in the Source Data file.

Research involving human participants, their data, or biological material

Policy information about studies with human participants or human data. See also policy information about sex, gender (identity/presentation), and sexual orientation and race, ethnicity and racism.

Reporting on sex and gender	N/A
Reporting on race, ethnicity, or other socially relevant groupings	N/A
Population characteristics	N/A
Recruitment	N/A
Ethics oversight	N/A

Note that full information on the approval of the study protocol must also be provided in the manuscript.

Field-specific reporting

Please select the one below that is the best fit for your research. If you are not sure, read the appropriate sections before making your selection.

- [x] Life sciences
- [ ] Behavioural & social sciences
- [ ] Ecological, evolutionary & environmental sciences

For a reference copy of the document with all sections, see nature.com/documents/nr-reporting-summary-flat.pdf

Life sciences study design

All studies must disclose on these points even when the disclosure is negative.

Sample size	All available samples were analyzed in this study
Data exclusions	No data was excluded
Replication	Viral titers, fluxes and lung pathology measurements, antibody analysis were performed twice by independent researchers in the lab. The replications were successful. The variation in duplicate experimental runs was <10% for replicates.
Randomization	Four- to 6-week-old female BALB/c mice of 5 mice/group were randomized to be injected with different MABs and infections with either RSV-A2 or RSV-B1. The different samples collected from these mice were were also randomly allocated per treatment group for various analyses.
Blinding	Experiments were performed by different investigators, who were blinded to sample identity.

Reporting for specific materials, systems and methods

We require information from authors about some types of materials, experimental systems and methods used in many studies. Here, indicate whether each material, system or method listed is relevant to your study. If you are not sure if a list item applies to your research, read the appropriate section before selecting a response.

Materials & experimental systems

n/a	Involved in the study	
[ ]	[x]	Antibodies
[ ]	[x]	Eukaryotic cell lines
[x]	[ ]	Palaeontology and archaeology
[ ]	[x]	Animals and other organisms
[x]	[ ]	Clinical data
[x]	[ ]	Dual use research of concern
[x]	[ ]	Plants

Methods

n/a	Involved in the study	
[x]	[ ]	ChIP-seq
[x]	[ ]	Flow cytometry
[x]	[ ]	MRI-based neuroimaging

Antibodies

Antibodies used	Unique panel of RSV anti-G MAbs were produced in our lab for studies described in this manuscript. Synagis (Palivizumab; anti RSV-F) was purchased from the NIH Pharmacy (Cat No- 1000509). HRP conjugated Goat anti-human IgG (Fc) antibodies were purchased from Jackson ImmunoResearch (Cat number 109-035-098). Both commercial antibodies were used at 1000-fold dilution.
Validation	Validation of mouse MAbs binding to RSV-G was performed in the lab for specificity, linearity, precision, accuracy, robustness and

technical reproducibility for binding to G protein to RSV-A2 and RSV-B1 strains using ELISA. Commercially purchased Palivizumab from NIH Pharmacy (Cat No- 1000509) and HRP conjugated Goat anti-human IgG (Fc) antibodies from Jackson ImmunoResearch (Cat number 109-035-098) were validated by the respective manufacturers using ELISA and immunoelectrophoresis.

Eukaryotic cell lines

Policy information about cell lines and Sex and Gender in Research

Cell line source(s)	293-Flp-In cell line (Cat. No. #R75007) were obtained from ThermoFisher Scientific. A549 cells (Cat. No. #CCL-185) were obtained from the American Type Culture Collection.
Authentication	Cell lines were obtained fresh from commercial providers and grown as per manufacturer's instructions. None of the cell lines were authenticated by karyotyping or other genomic techniques.
Mycoplasma contamination	Negative for Mycoplasma
Commonly misidentified lines (See ICLAC register)	No misidentified cell lines were used in the study.

Animals and other research organisms

Policy information about studies involving animals; ARRIVE guidelines recommended for reporting animal research, and Sex and Gender in Research

Laboratory animals	Four- to 6-week-old female BALB/cAnNCr strain mice from Charles River Labs (code #555).
Wild animals	N/A
Reporting on sex	Sex of mice species were not considered in study design. Only female BALB/c mice were included in the study, based on experience with previous studies in the lab on RSV infection and previous vaccination and therapeutic studies have only used female BALB/c mice. Based on historical datasets and experience, we continued using the female mice for our RSV studies. It is expected that finding are applicable for animals of both sexes.
Field-collected samples	No field samples were collected. For BALB/c mice, 12 light/12 dark cycle, ambient temperature (70-75 F), and 45-55% relative humidity was used for housing as per National Institutes of Health (NIH) guidelines.
Ethics oversight	All animal experiments were approved by the U.S. FDA Institutional Animal Care and Use Committee (IACUC) under Protocol #2009-20. The animal care and use protocol meets National Institutes of Health (NIH) guidelines.

Note that full information on the approval of the study protocol must also be provided in the manuscript.

Plants

Seed stocks	N/A
Novel plant genotypes	N/A
Authentication	N/A
